# Optimizing treatment for depressed parents of children with emotional and behavioral disorders: A multi-site feasibility trial

**DOI:** 10.1371/journal.pone.0351733

**Published:** 2026-07-08

**Authors:** Brendan F. Andrade, Sabrina Brodkin, Aliza Israel, Tatiana Valverde da Conceicao, Madison Aitken, Tatiana Djurovic, Marco Battaglia, Anne-Claude Bedard, Anjali Suri, Darren Courtney, Hali Kil, Stefan Kloiber, Judith M. Laposa, Clement Ma, Simone N. Vigod, Peter Szatmari

**Affiliations:** 1 Campbell Family Mental Health Research Institute, Centre for Addiction and Mental Health, Toronto, Canada; 2 Department of Psychiatry, University of Toronto, Toronto, Canada; 3 Department of Applied Psychology and Human Development, Ontario Institute for Studies in Education, University of Toronto, Toronto, Canada; 4 Department of Psychiatry, Women’s College Hospital, Toronto, Canada; 5 Department of Psychology, Faculty of Health, York University, Toronto, Canada; 6 Department of Medicine, Royal College of Surgeons in Ireland, Dublin, Ireland; 7 Department of Psychology, Simon Fraser University, Burnaby, Canada; 8 BC Children’s Hospital, Vancouver, Canada; IU Internationale Hochschule GmbH, GERMANY

## Abstract

**Background:**

Behavioral parent training (BPT) programs are effective for reducing disruptive behavior of many children with emotional and behavioral disorders (EBD) and improving skills and competencies of their parents. However, these interventions are not sufficiently effective for parents who themselves have depression. This study assessed the feasibility of the Addressing Depression and Positive Parenting Techniques (ADAPT) Program—a novel BPT that includes aspects of cognitive behavioral therapy and dialectical behavior therapy. We identified whether recruitment was possible, attendance was acceptable, participants were satisfied, and facilitators adhered to the protocol. Additionally, we measured trends in changes in parent and child outcomes with intervention.

**Methods:**

Participants were 28 parents and their children with EBD, referred to two hospital-based clinics. Parents were eligible if they (1) had a child aged 6–10 experiencing emotional and behavioral difficulties and (2) were experiencing elevated depressive symptoms. ADAPT took place virtually over 10 weeks. Participants completed measures before, weekly and after treatment. Clinicians recorded attendance and completed weekly treatment fidelity checklists.

**Results:**

We found that recruitment was possible, the intervention showed high parental satisfaction, and facilitators reported high program fidelity. Further, secondary outcome measures showed evidence of sensitivity to change in the intended direction.

**Conclusion:**

Given that a priori progression criteria were met and we found preliminary indication of efficacy, we conclude that an RCT of ADAPT to determine efficacy is warranted.

## Introduction

Emotional and behavioral disorders (EBD) affect between 5–15% of children and are associated with impaired social development and quality of life for children [1]. These children experience elevated levels of difficulty with emotion and behavior regulation resulting in both internalizing and externalizing challenges [2,3]. Behavioral Parent Training (BPT) is amongst the most well-established and evidence-based treatments for children with EBDs [4]. Unfortunately, BPT does not meet the needs of all, with up to 40% of parents and children not sufficiently benefiting from this intervention [5].

Parental depression at baseline appears to be predictive of poorer BPT outcomes [6,7]. Parents with elevated symptoms of depression do not appear to sufficiently gain key skills from BPT, and their children do not show adequate reduction in challenging behaviors [8]. This is an important issue to address because parents of children with EBDs have elevated rates of depression compared to the general population, with as many as 50% experiencing symptoms of anxiety or depression [7,9,10]. As such, improving the effectiveness of BPT in the context of parental depression could have large potential for a positive impact on short- and longer-term parent and child functioning.

A limited body of research has identified factors that may partly explain why some parents with depression may not benefit from BPT. One factor that has been proposed to negatively impact parent engagement in BPT is low “readiness.” Readiness refers to a parent’s understanding of the treatment benefits and barriers and willingness to learn and implement new skills and strategies [11]. Specifically, parent’s with depression tend to report lower motivation and readiness to engage in treatment [12]. Given that BPT relies heavily on consistent skills practice, homework completion, and in-session participation, reductions in engagement can disrupt the acquisition and consolidation of parenting skills, negatively influencing outcomes [4].

Additionally, it is well established that cognitive processes differ in individuals experiencing depression compared to those who aren’t depressed, evidenced by elevated negative beliefs about themselves and others [13]. For parents who are depressed, their negative perceptions about the causes of their children’s behavioural challenges, termed ‘attribution biases’, appear to be related to intervention outcomes [14]. Parents with depression show elevated negative attribution biases [15] a tendency to interpret the cause of their child misbehavior as intentional, stable, and internally driven, rather than to situational or developmentally normative causes. As a result, parents with depression may have more difficulty accepting BPT as a logical form of intervention for them and may view it as a mis-match for their child’s challenging behaviors. Further, because of this perceived mismatch, depressed parents may not appreciate the value of implementing BPT principals, leading to non-participation, treatment drop-out and diminished treatment response.

Finally, emotion dysregulation, evidenced in depressed parents of children with EBD, may be a factor contributing to poorer BPT outcomes [10]. Implementing BPT strategies often requires a high level of emotion management from the parent (i.e., the ability to tolerate distress, inhibit reactive responses, and respond to child behavior in a consistent and regulated manner). Parents with depression experience heightened emotional reactivity and difficulties with emotion management [16]. Additionally, research highlights the importance of adult modelling of effective emotion regulation (ER) to enable the development of these skills in children [10]. If depressed parents have difficulties with emotion regulation, this may impact not only use of BPT strategies, but also children’s opportunities to internalize these skills through observation.

Addressing Depression and Positive Parenting Techniques (ADAPT) is a novel treatment developed for parents of children with EBDs who experience depression themselves. ADAPT builds on evidence-based BPT by integrating elements of motivational interviewing to increase parental motivation/readiness, cognitive behavioural therapy to address parental negative attribution bias, and dialectical behavioural therapy to build parental ER skills. In the present study the ADAPT program was implemented as part of a non-randomized, external pilot trial at two major hospital sites with the aim of evaluating its feasibility in preparation for a future randomized controlled trial (RCT) to determine program efficacy. External pilot trials are a type of feasibility study aimed specifically at determining whether a large scale RCT can and should be done [17,18]. These trials are also helpful for highlighting key considerations that need to be made before embarking on the RCT, a more costly and time-consuming project. The pilot trial is labeled “external” as the outcome data is not used for the RCT. Further, the focus is not on outcomes but rather on the ability to run the trial and treatment program. In line with the recommendations of Mellor and Colleagues [19] for external pilot trials, we laid out the following progression criteria a priori:

1. Recruitment of parents with elevated depression who have children with EBD will be possible based on our ability to fill multiple groups (4–6 groups across 2 sites).
2. The intervention will be acceptable based on attendance (≥ 70% of sessions) and participant satisfaction (≥ 70% overall satisfaction).
3. There will be a high rate of adherence to the study protocol by clinicians (≥ 70% of session content completed).

Secondarily, we explored whether the selected outcome measures were sensitive to change and whether positive trends in parent and child outcomes were observed.

## Methods

In addition to Mellor et. al’s framework, we used the 2010 CONSORT Extension for feasibility trials [20] and the TREND statement for nonrandomized intervention evaluations [21] as a reporting guide. The study was approved by the Centre for Addition and Mental Health Research Ethics Board (CAMH REB) on September 30^th^, 2019. The CAMH REB is qualified through the CTO REB Qualification Program and is registered with the US Department of Health and Human Services (DHHS) Office for Human Research Protection (OHRP). The study was preregistered (ClinicalTrials.gov ID: NCT04298437).

### Participants

The primary group of participants in the present study were parents who presented to two hospital clinics in Toronto for their child’s emotional or behavioral difficulties. Their children represent a secondary group of participants, and the group facilitators represent a tertiary group of participants. Recruitment took place between August 4^th^, 2022 and February 2^nd^, 2024, with the aim of filling multiple groups across settings.

A parent was eligible if they: (1) were experiencing mild to moderate elevations in depression symptoms indicated by a score from 5–15 on the Patient Health Questionnaire-9 (PHQ-9: Kronke et al., 2001), (2) had a child who was experiencing elevated emotional and/or behavioral difficulties, indicated by a score > 92nd percentile on the Total Difficulties and/or Conduct Problem scale of the Strengths and Difficulties Questionnaire (SDQ: Goodman, 1997), (3) were able to communicate in English, and (4) were willing to participate in group treatment. A parent was excluded if their child was diagnosed with Autism Spectrum Disorder or if they were experiencing very elevated levels of depression symptoms (PHQ-9 score > 14), active substance use disorder, psychosis, or suicidal ideation. Once eligible to participate, parents were permitted to bring a partner to the group (i.e., their husband or wife). The partner was not required to meet the inclusion criteria.

The group facilitators were trained clinicians from a range of disciplines (i.e., psychologists, psychiatrists, psychology graduate trainees, social workers, and child and youth workers). Each group was co-facilitated by up to 3 clinicians, such that someone was able to adequately monitor the fidelity of each section of the group.

### Procedure

One parent per family began the study with a screening call to ensure they met inclusion criteria. During this call, a trained senior lab member reviewed the consent form for the study, explaining the purpose of the research study, the risks and benefits of participating, the expected duration of participation, the responsibilities and compensation for participating, confidentiality and privacy, and that all participation is completely voluntary. After the Consent Form had been reviewed in great detail, all participants were asked to consent using electronic consent procedures. Electronic consent was obtained from participants using the REDCap e-Consent framework developed by CAMH.

If eligible and consenting, the parent attended an in-person assessment with their child, where questionnaires and lab-based tasks were completed. As a single-arm, non-randomized pilot trial, all eligible parents were then invited to participate in the ADAPT program. At this stage, if a second parent was interested in joining the treatment program they were included. The program begins with an initial individual engagement session followed by 9 virtual group sessions. During the 10 weeks of treatment, parents completed weekly measures (detailed below) and satisfaction surveys, and clinicians completed attendance and fidelity checklists.

#### Intervention.

The ADAPT program is a manualized treatment with 10 virtual sessions (Table 1). Unlike most BPT groups, the first session is an individual meeting between a clinician and parent(s) incorporating a motivational interviewing framework. The goal of this session is to build rapport with the parent, learn about their perspective of their child’s emotional and behavioral needs, and discuss treatment expectations, goals, and program content. The overall purpose of the first session is to engage the parent(s), build a working alliance, and leave the parent with the expectation that ADAPT may be helpful. For the following 9 group sessions, participants are provided with weekly booklets, including activities to be completed during the group and at home. Each session begins with agenda setting and a review of the previous week’s home practice, then clinicians work through the session content and end by assigning home practice. Weeks 2–5 focus on helping parents understand factors that may underlie their child’s emotional and behavioral challenges, build their own ER and distress tolerance skills, and learn alternative and more helpful ways of thinking about the potential causes of their child’s emotional and behavioral problems. Then, in weeks 6–10, parents learn key BPT strategies like validation, praise, and effective instructions. ADAPT concludes with parents developing a personalized action plan that combines parent- and child-directed strategies to promote continued practice and generalization of skills.

**Table 1:** ADAPT Program Content.

Session #	Session Content
1	**Individual Engagement Session** – planning and goal setting
2	**Child Learning Targets** – underdeveloped skills and opportunities for growth
3	**Your Thoughts** – helping parents understand helpful and unhelpful thinking and a Wise Mind approach to parenting
4	**Your Emotions** – helping parents develop helpful self-regulation strategies
5	**Understanding Child Emotion and Behavior** – helping parents understand the factors that contribute to their child’s emotion and behavior difficulties
6	**Validation** – helping parents develop strategies to validate their own and their child’s emotion
7	**Play, Special Time and Praise** – helping strengthen parent-child relationships
8	**Giving Effective Instructions, Rules and Expectations** – helping parents develop realistic home-based structures
9	**Managing Behavior Difficulties** – helping parents develop strategies to limit their children’s challenging behavior
10	**Family Problem Solving** – helping parents develop a problem-solving approach and personalized action plan that

### Measures

#### Attendance, satisfaction and fidelity measures.

At the beginning of each ADAPT treatment session, a facilitator tracked the attendance of each member of the group, noting any absence, lateness, and reasons for non-attendance. Regarding fidelity, throughout the treatment, weekly meetings between the facilitators occurred during which a fidelity checklist was completed. The fidelity checklist asked facilitators to indicate whether the session-specific goals were attained and whether the session objectives were not covered, partially covered, or fully covered. In the case that a goal or objective was not met or covered, facilitators reported why this occurred. Lastly, following each session, parents were administered a satisfaction questionnaire, which asked them to indicate how satisfied they were with the session ranging from ‘very dissatisfied’ to ‘very satisfied’. Parents were also asked to note what they liked the most and least about the session, and whether they had suggestions for improvement. These qualitative findings will be presented in a subsequent paper.

#### Child screening & outcome questionnaires.

Two parent-report questionnaires were used to evaluate child emotions and behavior before, during, and after the ADAPT program. The Strengths and Difficulties Questionnaire (SDQ) [22] was used at baseline and both the conduct problem and total difficulties scores were used for screening purposes. The Behavior and Feelings Survey (BFS) [23] was administered weekly, and the total score was used to evaluate change in children’s internalizing and externalizing psychopathology.

The Strengths and Difficulties Questionnaire (SDQ) is a widely used and well validated tool to evaluate positive and negative attributes of a child’s behavior and emotions [24]. Previous research looking at the total difficulty scale demonstrated acceptable internal consistency (α = .73) [25], and test–retest reliability (r = .62) [26]. The parent report form of the SDQ asks parents to rate statements such as ‘often loses temper’ and ‘kind to younger children’ in reference to their child’s behavior over the last six months ranging from 0 = not true, 2 = somewhat true, or 3 = certainly true. Overall, it evaluates emotional symptoms, conduct problems, hyperactivity/inattention, peer relationship problems, prosocial behavior, and total difficulties.

The BFS is a psychometrically sound tool, developed specifically for efficient progress monitoring during treatment, with excellent internal consistency (α = .92), and strong test-retest reliability (r = .85) [23]. The measure evaluates parent’s perceptions of how challenging children’s thoughts, feelings, and behaviors have been over the past week, and asks parents to describe “how much their child had each of the following problems during the past week?” and then asks them to rate each item on a scale from 0 = not a problem and 4 = a very big problem. The BFS is split into two sections – a ‘thoughts and feelings’ section that evaluates problems such as “felt sad” and “felt nervous or afraid”, and a ‘behavior and conduct’ section that evaluates problems such as “Refused to do what adults told them to do” and “Argued with people”.

#### Parent screening & outcome questionnaires.

Three questionnaires were administered to assess parents’ mood and behavior. Specifically, the Parent Health Questionnaire 9 (PHQ-9) [27] was used to assess parental depression symptoms at baseline. The Depression, Anxiety and Stress Scale (DASS-21) [28] was administered weekly to evaluate parent stress in the past week. Additionally, the Alabama Parenting Questionnaire (APQ) [29] was administered weekly to measure parenting behaviors that were identified as relevant for the treatment of EBDs.

The PHQ-9 is a brief, accessible screener for depression symptoms that has been well validated for use in primary care and as a progress monitoring tool, with a good internal consistency (α = .87) [30]. It asks parents “over the past 2 weeks, how often have you been bothered by any of the following problems?” and provides them with the following options: 0 = not at all, 1 = several days, 2 = more than half the days, and 3 = nearly every day. This measure evaluates symptoms such as “little interest or pleasure in doing things”, “poor appetite or overeating”, and “thoughts that you would be better off dead or of hurting yourself in some way”. It also asks parents “how difficult have those problems made it for you to do work, take care of things at home, or get along with other people”, ranging from not difficult at all to extremely difficult. A score of 0–4 indicates minimal or no depression, 5–9 indicates mild depression, 10–14 indicates moderate depression, 15–19 indicates moderately severe depression, and 20–27 indicates severe depression.

The DASS-21 is a valid and reliable tool that evaluates parent depression, anxiety, and stress symptoms [31]. In the context of the present trial, we included the stress subscale, which has a strong internal consistency (α = .90) [31] and measures symptoms related to difficulty relaxing, nervous arousal, and being easily upset/agitated, irritable/over-reactive, and impatience. Parents were asked to rate how often they experienced certain symptoms within the last week on a scale from 0 = never, 1 = sometime, 2 = often, and 3 = almost always.

### Analysis

All data analysis was completed in SPSS (Version 29). Descriptive statistics were generated to summarize participant attendance, clinician fidelity, and participant satisfaction. Paired-sample *t*-tests were used to analyze pre- to post-treatment changes in parent stress, parenting behaviors, and child internalizing and externalizing psychopathology for participants meeting study inclusion criteria (i.e., depressed parents, not their partners) and for whom pre- *and* post-treatment data was available (N = 22). Specifically, we analyzed data from the pre-treatment assessment and from after the last treatment session. A formal sample size calculation was not conducted, as the purpose of this study was to evaluate feasibility parameters consistent with guidance on pilot trials [17,20]. We report effect sizes (Cohen’s d) and mean differences, with a 95% confidence interval, instead of p-values, to reflect the aims of the paper (i.e., to primarily understand the feasibility and secondarily to assess measurement sensitivity to treatment and trends in the data).

## Results

**Fig 1:**
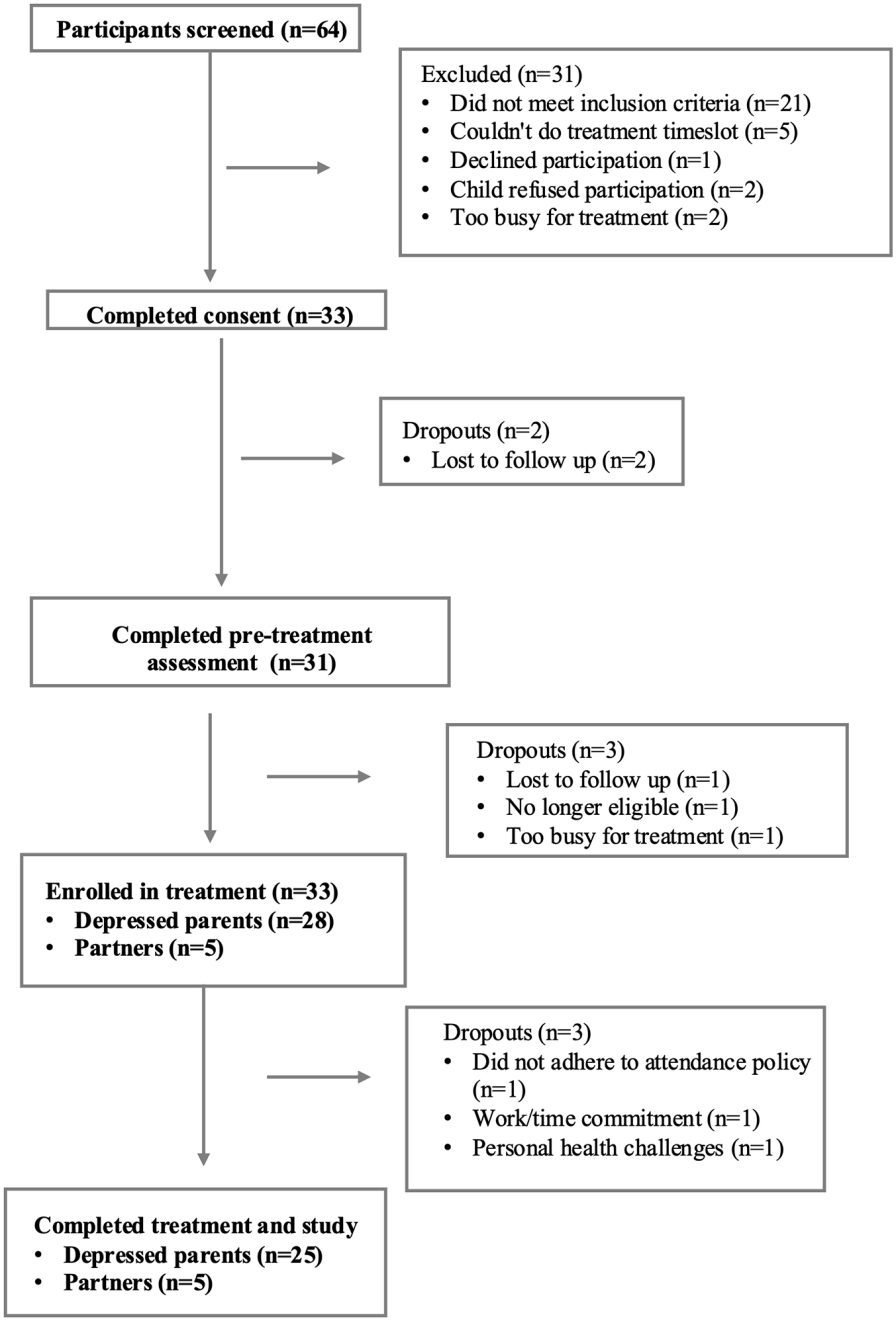
CONSORT Diagram.

Recruitment for the ADAPT program, described in Fig 1, was deemed possible as we were able to fill five groups. The final sample included 28 parents with elevated levels of depression and 5 of their partners (33 total individuals in the group). There were 28 children in the sample and 10 clinicians. The parents were between the ages of 31 and 56 (mean = 44), and 26 of the primary participants (parents with elevated levels of depression) were female. The children were between the ages of 5 and 10 years (mean = 8.3), and 22 of the children were male. The table below provides socio-demographic information for the eligible parent participants and their children (Table 2).

**Table 2:** Socio-demographic information.

Child Characteristics	
Age (M ± SD)	8.4 ± 1.5
Gender, n (%)	6 female (21%)
**Parent Characteristics**	
Gender, n (%)	26 female (90%)
Race/Ethnicity, n (%)	
European Origins	21 (72%)
Caribbean Origins	2 (6%)
Other North American Origins	2 (6%)
Other	3 (10%)
Age (M ± SD)	44.00 ± 5.89
Education	
Completed post-secondary	22 (76%)
Some post-secondary	3 (10%)
High School	3 (10%)
Family Income	
$150,000 or more	11 (38%)
$120 000 - $149 999	0 (0.0%)
$90,000–$119,999	4 (14%)
$60,000–$89,999	3 (10%)
$30,000–$59,999	3 (10%)
$15,000–$29,999	3 (10%)
Prefer not to answer	3 (10%)
Do not know	1 (3%)
**Screening Variables**	
PHQ-9 Score (Parent), M ± SD	12.6 ± 4.9
SDQ Conduct Problems (Child), M ± SD	4.9 ± 2.4
SDQ Total Difficulties (Child), M ± SD	21.5 ± 3.7

Note. A PHQ-9 score of 0–4 represents no significant depressive symptoms; 5–9, mild symptoms; 10–14, moderate symptoms; 15–19, moderately severe symptoms; and 20–24, severe symptoms. An SDQ Conduct Problem Score of 0–2 falls in the “Normal” range, a score of 3 falls in the “Borderline” range and a score of 4–10 falls in the “Abnormal” range. An SDQ Total Difficulties score between 0–13 falls in the “Normal” range, a score between 14–16 falling in the “Borderline” range and a score between 17–40 falls in the “Abnormal” range.

The attendance rate was deemed acceptable at 85% overall for the 10 sessions, surpassing our goal of 70%. Additionally, the intervention was considered acceptable based on weekly satisfaction ratings, with 98% of participant responses indicating that they were Very Satisfied or Somewhat Satisfied (Very Satisfied = 71%, Somewhat Satisfied = 27%) with the session that week. The group facilitators met the criteria for fidelity, adhering closely to the protocol and reporting on average, 94% of objectives and 99% of goals were met on fidelity measures. The results of the paired-sample *t*-tests on preliminary outcome data can be seen in the table below (Table 3).

**Table 3:** Outcome Data.

Measure (Subscale)	Mean Pre	Mean Post	Mean Difference (95% Confidence Interval)	Cohen’s *d*^a^
DASS-21 (Stress)	11.45	8.95	2.50 (0.07, 4.93)	0.48
APQ (Inconsistent Discipline)	17.35	15.47	1.88 (0.62, 3.14)	0.77
APQ (Positive Parenting)	25.06	26.06	−1.00 (−2.19, 0.19)	−0.43
APQ (Poor Monitoring)	13.35	11.76	1.59 (0.06, 3.12)	0.53
BFS (Total Difficulty)	25.38	17.29	8.09 (5.28, 10.90)	1.22

Note. Cohen’s d for paired samples was calculated by dividing the mean of the paired difference scores by the standard deviation of the paired difference scores. A positive mean difference and Cohen’s d value indicates a decrease in the construct over time, while a negative mean difference and Cohen’s d value indicates an increase in the construct over time.

^a^0.2-0.49 = small, 0.5-0.79 = medium, 0.8+ = large.

## Discussion

The present study evaluated the feasibility, acceptability and outcome trends of a novel BPT, called ADAPT, tailored to the emotional and cognitive needs of depressed parents who have children with EBDs. Specifically, past research had suggested parent readiness, attribution biases, and emotion dysregulation as key mechanisms contributing to unsatisfactory treatment benefits for depressed parents. Accordingly, content within the novel ADAPT program was developed with these mechanisms in mind and incorporates components from motivational interviewing, cognitive behavioral therapy and dialectical behavior therapy to enhance BPT to better fit the emotional and behavioral needs of parents with depression who have children with emotional and behavioral difficulties. We found that all a priori progression criteria were met – recruitment was possible, ADAPT was acceptable based on attendance and satisfaction, and treatment fidelity was high.

Specifically, we successfully filled five ADAPT groups across two major hospitals in a large urban center. Moreover, we had high retention rates across the program and parent participants reported high rates of satisfaction with ADAPT. Engagement (i.e., attendance, retention, and adherence) in BPT has been a critical and longstanding issue, with estimates of about 20 percent of parents who enroll in BPT dropping out [32]. The high attendance for ADAPT suggests that integrating motivational interviewing, CBT, and DBT strategies to target challenges with engagement, unhelpful thinking and emotion regulation in depressed parents, may improve their participation and potential benefits from this enhanced BPT. This is aligned with previous research examining the cause of low engagement and high dropout rates identified that parents were more likely to remain in treatment if they understood how the BPT would address their current needs [32], and if the treatment directly addressed their motivation and maladaptive cognitions around potential improvement [33]. However, it is noteworthy that the intervention was delivered virtually, which may have been a contributing factor to the high attendance and low dropout rates. The impact of virtual delivery on engagement remains unclear and has been inconsistently measured [34]. Notwithstanding, it is possible that reduced logistical barriers (e.g., transportation, childcare) contributed to retention, rather than intervention content alone, and this should be considered in a future RCT.

Clinicians reported fidelity to the program content and agreement that they could work through the program components within the designated timeframe, and that completing session objectives and therapeutic goals over the course of each group was possible. This finding provides evidence that the structured training for facilitators sufficiently prepared them for implementation of ADAPT. Further high levels of fidelity provide indication that the session content was appropriately paced, and the materials (i.e., manuals and slide decks) were useful in guiding each session. From an implementation science perspective, clinician experience is a critical determinant of a program’s feasibility and sustainability. Interventions are only viable at scale if they can be delivered with fidelity in routine care settings without placing undue burden on providers [35,36]. These strong findings suggest that ADAPT is well-designed for clinician delivery and suitable for broader dissemination. However, these assertions need to be tested through a subsequent randomized trial.

Investigation of trends in outcomes showed small to large effects in the expected directions, suggesting that the measures are sensitive to change and the program may have positive effects on parents and children. Specifically, the treatment impacted parenting strategies, with a decrease in parental inconsistent discipline. Positive parenting showed a slight increase and poor monitoring showed a moderate decreases following treatment. There was also a small decrease in parent stress following intervention. Notably, there was a large decrease in child emotional and behavioral difficulties reported by parents following treatment. These findings are aligned with large-scale reviews and meta-analyses of BPT programs and hold promise for a larger-scale trial to determine the efficacy of ADAPT [4,37,38] (Kaminski et al., 2024; Weber et al., 2019; Mingebach et al., 2018). However, these findings should be interpreted with caution given that outcome data was collected from a single reporter and was only collected immediately after the group ended.

Understanding the efficacy of the ADAPT program on a larger scale, through an RCT, will help illuminate whether directly targeting factors such as parent readiness for treatment, negative attribution biases, and parental emotion dysregulation, could improve treatment outcomes for depressed parents and their children with EBDs compared to standard treatment. If the RCT were to be effective, this would be a promising advancement in the treatment of EBDs.

There are limitations of the present study that should be considered. First, the sample was not representative of the general population, with a vast majority of the participants being female and with higher household incomes. ADAPT is intended as a general intervention for depressed parents regardless of gender. A future RCT may examine gender related differences in engagement and outcomes may be important. Overall, recruiting a more diverse group of parents for a future RCT will be important to facilitate generalizability of findings. A second limitation is that this study relied on parent report questionnaires as the only data source. In a future trial, it may be important to obtain multiple sources of data, including more objective measures and questionnaires from alternative reporters (i.e., observational tasks, teacher questionnaires). Novel approaches like Ecological Momentary Assessment data collection could be considered as an approach to measure BPT skill use. Third, future trials could measure the proposed mechanisms of change (i.e., readiness, attribution biases, and emotion dysregulation) more directly to determine whether the program is adequately targeting these areas, and whether that contributes to better outcomes for depressed parents. Standardized rating scales and structured interviews could be used to gather data on each variable at baseline, throughout treatment, and after treatment. Obtaining direct measures of these key mechanisms, and monitoring for change over the course of the treatment, could also help elucidate which aspects of the program are most impactful (i.e., the initial engagement session, the CBT session, the DBT content etc.).

In sum, given the success of the pilot trial, we conclude that, following treatment development guidelines, a sufficiently powered RCT to determine efficacy is warranted. This RCT should compare ADAPT to standard BPT or treatment-as-usual with an adequately powered sample, and include multiple informants, observational data, and longitudinal follow-up. If an RCT were to establish that the ADAPT program can enhance treatment outcomes for depressed parents of children with EBDs this could have a meaningful impact on quality of life for a substantial number of families.

## Supporting information

S1 File ADAPT Study Protocol. The attached document is the protocol for the ADAPT study.
(DOCX)

S2 File CONSORT Checklist. This is the completed CONSORT checklist for pilot and feasibility trials.
(DOC)
