# Supplementary material for: Optimizing treatment for depressed parents of children with emotional and behavioral disorders: A multi-site feasibility trial
Source: PLoS One. 2026 Jul 8;21(7):e0351733. doi: 10.1371/journal.pone.0351733 (PMC13345296; doi:10.1371/journal.pone.0351733)
Supplement: S1 File — The attached document is the protocol for the ADAPT study. (DOCX) [file pone.0351733.s001.docx]

**Protocol**

TITLE: Optimizing Treatment for Parents of Children with Emotional and Behavioural Problems (OPTED)

PRINCIPAL INVESTIGATOR: Dr. Brendan Andrade, Ph.D., C.Psych

INVESTIGATOR CONTACT: Centre for Addiction and Mental Health

Child, Youth and Emerging Adult Program

Intergenerational Wellness Building

80 Workman Way, Room 1109

Toronto, Ontario, M6J 1H4

(416) 535-8501 ext. 33642

Fax: (416) 979-4685

[Brendan.andrade@camh.ca](mailto:Brendan.andrade@camh.ca)

PRINCIPAL INVESTIGATOR: Dr. Aliza Israel

INVESTIGATOR CONTACT: Women’s College Hospital

Child & Adolescent Psychiatry

Reproductive Life Stages Program

76 Grenville Street

Toronto, Ontario, M5S 1B2

(416) 323-6230

Aliza.israel@wchospital.ca

Background

Children with emotional and behavioral disorders (EBD) experience disproportionate social, family and academic impairment and are 2 to 5 times more likely to develop a severe mental illness in adolescence and adulthood [1-3]. Similarly, parents of children with EBD experience disproportionate stress and much research has documented the close association between parental depression and the emergence and maintenance of childhood EBD [4-7]. Designing targeted and effective intervention for parents of children with EBD that take into account parents’ mental health needs is necessary. However, and of pressing concern, first-line Behavioral Parent Training (BPT) treatments for parents of children with EBD are not tailored to parents and approximately 40% of parents and children fail to sufficiently benefit [8, 9]. Findings from our group, and emerging international research, point to key domains for innovation.

First, our published findings show that parents of children with EBD are heterogeneous with regards to their mental health needs, with over 50% of parents themselves having elevated emotional difficulties (i.e., mood or anxiety problems)[10]. These parents have difficulties regulating their emotion and behavior during stressful interactions with their children. Further, our published preliminary data show that parental emotional difficulties are associated with less benefit from BPT, evidenced by less reduction in children’s EBD and less increase in positive parental competencies and skills compared to parents of children with EBD without emotional difficulties [10].

Second, findings from Dr. Andrade’s (PI) ongoing CIHR funded clinical trial to personalize psychosocial intervention for children with behavior disorders show that parent cognitions about the cause of their child’s behavioral difficulties are associated with BPT outcomes. Parents who attribute the cause of children’s EBD to negative internal and stable aspects of their child appear less amenable to shifting their parenting behavior, possibly because they perceive that their children are intentionally creating problems through their behavior[11]. Biased thinking in these parents, which parallel dysfunctional cognitions of depressed adults, appear to be another factor limiting BPT outcomes. These lines of evidence suggest that innovative and tailored assessment and treatment approaches that takes into account parental depression, associated emotion dysregulation and biased cognition may improve outcomes.

Objectives

The central **objectives** of this research are: **(1)** To evaluate the feasibility of recruitment for this type of treatment based off the findings of the Needs Assessment Survey. *We hypothesize that recruitment will be feasible and that there will be enough families seeking out this type of service.* (2) To determine objective emotional, cognitive and behavioral markers that may improve the precision of clinical assessment. We will conduct a mixed-methods cross sectional study to compare parent and child emotional regulation, cognition (i.e., thinking patterns) and behavior during a validated and stress inducing parent-child interaction task between depressed and non-depressed parents and their children with EBD. We will also use an ecological momentary assessment method to gather daily ratings of parent observations of their own and their child’s emotional-behavioural functioning. Current best practice assessment procedures for EBD solely rely on retrospective behavioral ratings that may be biased based on parent’s perceptions and their own mental health difficulties. Inclusion of objective measures, including markers of parent and children’s emotion regulation and cognition into assessment, and temporally close ratings of emotional-behavioural functioning, may strengthen diagnostic precision and inform targeted treatment selection. *We hypothesize that depressed parents will show less positive and more negative emotion and greater biased cognition during the dyadic task compared to non-depressed parents*. *Further, we hypothesize that greater biased parental cognition will escalate the negative effect of parental depressive symptoms on child emotional-behavioural daily functioning.* (3) To evaluate the feasibility and acceptability of a novel targeted treatment for depressed parents of children with EBD. To date, we have developed a protocol based on an untested treatment and have developed processes for identifying and recruiting depressed parents. We now aim to conduct a two-site (CAMH-Women’s College Hospital; WCH) open-label pilot study to provide meaningful data regarding *feasibility and acceptability* of the treatment and *adherence* to the study procedures. We will use the results to make key modifications to study procedures and the treatment itself to increase the success of *a future RCT to test efficacy*. *We hypothesize that recruitment will be feasible, that the intervention will be acceptable, and that there will be a high rate of adherence to the study protocol*. We will seek funding for a subsequent RCT of the study protocol if we meet the following criteria: 1) recruitment of 6 - 8 depressed parents for each pilot group; 2) > 70% of parents attend > 70% of the sessions in each pilot group; 3) > 70% of parents report acceptability of the group; 4) > 70% clinician adherence to treatment assessed by review of coded session checklists; and 5) > 70% of participants provide follow-up data for analysis. *Following established CONSORT guidelines for feasibility trials[12], a control group is not indicated given that program acceptability and outcome data cannot be used to assess efficacy, nor to estimate sample size required for a future large-scale study*.

For the duration of the COVID-19 pandemic where restrictions are in place for in-person group treatments, we will administer an adapted virtual version of the program. The program lends itself well to virtual delivery and the overall program will not change as a result of the change in mode of delivery. As part of the Better Behaviours Service at CAMH, where the PI oversees and runs parent-group treatments the mode of delivery for all group-treatment programs is now virtual and it continues to meet the needs of parents.

Research Plan

The study will have three components. First, to determine the feasibility of recruitment procedures we will recruit 100 parents to complete a brief questionnaire to assess their treatment needs. Second, to determine objective emotional, cognitive, and behavioral markers we will recruit 80 parents (40 depressed; 40 non-depressed) and their children with EBD. Parents and children will complete the parent-child interaction task, measure of cognition detailed below, and daily ratings about their child’s behaviour and their own psychopathology during the 10 days following participation in the research assessment. For the third component, a subset of 20-40 depressed parents will be recruited for group treatment to allow for sufficient variability in acceptability evaluation of the treatment. We aim to recruit 6 - 8 parents (and their co-parents) per group for each of 3 consecutive groups that will run over the course of 2 year at the CAMH and WCH (target n=36). Assuming a drop-out rate of ~20% that is consistent with the PIs ongoing trials, this would allow us to collect data on intervention acceptability from >29 participants. Participants will be recruited from the Child Youth and Emerging Adult Program (CYEAP) and Mood and Anxiety Service (MAAS) at CAMH and Women’s Mental Health Program (WMHP), in which the Child and Family Psychiatry Program is embedded at WCH. The strong clinical and research project team will ensure feasibility. The PI is an expert in BPT and has led clinical trials in the CYEAP over the last 8 years that recruit approximately 100 parents and children with EBD annually. Co-Is (SK, JL) are clinician-scientists who provide clinical services and run depression groups in MAAS. Co-PI (AI) and Co-I (CP) are child psychiatrists who treat children whose parents have mental illness at WCH. Co-Is (DC, SR, MA) run DBT youth groups and parent groups in CYEAP. Co-I’s (SV, MB) are Leaders in their respective programs and will ensure trial implementation and recruitment.

Selection criteria

We will include depressed and non-depressed parents who meet the following criteria: 1) 18 years of age or above; 2) they have children aged 6-10 years; 3) the aforementioned children have clinically elevated EBD (Abnormal Range, 93^rd^ percentile) on the Conduct Problems or Total Difficulties Scale of the Strength and Difficulties Questionnaire; 4) Show clinical elevations for Depression on the Patient Health Questionnaire-8 (>9); 5) Capacity to tolerate a group context assessed by clinical interview. Non-Depressed parents will also be recruited for the assessment portion of the study if the meet the above criteria but show a Patient Health Questionnaire-8 score in the non-clinical range. Parents are excluded if: 1) They show active suicidal ideation, substance use disorder, or psychosis; 2) cannot communicate in English; or 3) Child previously diagnosed with ASD.

Data Collection/Measures

The needs assessment questionnaire will be completed anonymously by parents who self-select to participate in the online REDCap questionnaire. Advertisements for the needs assessment will include a QR-code that can be scanned to automatically redirect phones to the questionnaire on REDCap. The questionnaire will first open to a page that asks for consent from the participants (view Needs Assessment Consent). Parents will then need to click the ‘Yes, I consent’ button to proceed to the Needs Assessment Survey. The questionnaire will not ask any identifying personal health information. REDCap is a widely used electronic data capture system that is effective in maintaining online databases and is security encrypted to ensure data remains secure.

Standardized protocols for recruiting research participants will be used by a research assistant (RA) and trained students at each site. The RA will screen interested participants for eligibility to participate in the study. Dyadic parent-child interaction task and FMSS (described below) will be completed by trainees’ naïve to group membership. A Group Acceptability Questionnaire will elucidate participant views about the intervention. Measures of parent cognition, behaviour mental health and child behaviour and emotional functioning will also be collected (see Appendix for descriptions of measures). Questionnaire data will be collected via a secure link in RedCap emailed to parents. The daily questionnaires will also be collected via a secure, encrypted link in RedCap, but will be texted to parents’ mobile devices via Twilio. Twilio is a widely used third-party SMS provider integrated with the REDCap platform. Once the participant clicks on a link to the survey, they will fill out the REDCap survey on their mobile device. REDCap goes to great lengths to ensure that SMS records do not stay in Twilio's logs but are removed shortly after being completed. This is done for security and privacy concerns, in which survey participants' phone numbers do not get permanently logged on Twilio's servers but instead remain securely in REDCap.

Clinician fidelity to treatment will be evaluated using audit of sessions by trained staff. Treatment acceptability reported by participating parents will be assessed using semi-structured interviews over phone or in person (Appendix V). Each site will maintain their own participant decoders for the duration of the study.

In order to ensure consistency between the two sites (CAMH and WCH), we will use a shared REDCap Project to administer questionnaires to participants. The “Data Access Groups” feature on REDCap will be used at both sites. The project coordinator at CAMH will assign all members to a site – CAMH or WCH – and members will only be able to access data entered at their respective sites. The CO-PIs for the study along with the project coordinator and graduate student at CAMH and the project coordinator at WCH will have access to data entered both sites for data quality assurance, troubleshooting, and tracking purposes. Once the study is complete, de-identified data will be transferred over from WCH to the CAMH using CAMH secure file transfer. All study data will be held on CAMH servers for 25 years after transfer.

Parent-Child Interaction Tasks measure parent and child emotional and behavioral responses to problem-solving activities. On these tasks, parents and children collaboratively solve a series of standardized activities that require problem-solving, emotion regulation and behavioral regulation skills. They engage in two complex tasks on which they are required to cooperate and problem-solve, and end with a free-play situation. Completion of activities will be video recorded for later coding of parent and child positive affect (expression of enjoyment, warmth or enthusiasm), negative affect (expression of anger, frustration, disapproval) and verbalizations (positive and negative cognitions) using a validated Dyadic Parent-Child Interaction Coding System[13], the Parent-Child Interaction System [17], and the Cognitive Sensitivity Caregiver-Child Thin Slice Dyadic Interaction Coding System [18]. Parental cognitions about their child’s behavior will be assessed using the Five-minute Speech Sample (FMSS); a validated approach to obtaining *independently coded* ratings of parents thinking and emotion with respect to their children.

Five-minute speech sample (FMSS); a validated approach to obtaining *independently coded* ratings of parents thinking and emotion with respect to their children[14]. A shorter version of the FMSS will be administered to the child, referred to as the Three-Minute Speech Sample (TMSS), to obtain independently coded ratings of children’s perception of their parents [19].

Conflict Discussion Task: a widely used approach to learn about conflict resolution strategies employed by parents and their children, specifically designed to look at the level of conflict constructiveness by looking at the display of positive and negative behaviours [20].

Addressing Depression and Positive Parenting Skills (ADAPT)is a targeted treatment adapted by the Co-PI (BA) based on the Parenting a Child Who Has Intense Emotions program[15]. The treatment is designed to be administered using two modes of delivery – in-person group sessions and virtual group sessions. The treatment has modules that help parents build emotion regulation skills, distress tolerance skills and helpful ways of thinking about their parenting. The program also contains components of standard BPT, including strategies to build family structure, routines and predictable parenting responses to children’s behavioral challenges. DBT is an effective intervention for the treatment of adults and youth with dysregulated emotion and behavior. However, ADAPT is a novel adaptation of this evidence-based approach and research is needed to establish its feasibility, and later its effectiveness, for depressed parents who show elevated levels of dysregulated emotion and biased cognition[16]. Two trained clinicians facilitate each group; parents have 1 private pre-group engagement session to familiarize them with the ADAPT group format and strengthen motivation for treatment.

For program development and quality improvement, co-investigators, team clinicians, and other stakeholders will be approached to provide feedback on our program via REDCap surveys. These questions will address group content, flow, clarity, presentation/delivery and relevance; and feedback will be used to improve the program.

During the COVID-19 pandemic, families will be invited to complete a virtual pre-assessment appointment to discuss the study and acquire consent, after which a few preliminary tasks will be completed. Depending on the family’s potential technology-related barriers, and the child’s ability to participate in a virtual assessment, families will be given three options: 1) an in-person assessment to complete all tasks once COVID restrictions are lifted (apart from the REDCap questionnaires) lasting 90 minutes; 2) a mixed virtual and in-person assessment (45 minutes) where some tasks will be completed virtually while others will be completed in person, once COVID restrictions have lifted; 3) a completely virtual assessment for those who do not feel comfortable with attending in-person (due to either COVID or other reasons).

The ADAPT Treatment Program will be offered virtually for the duration of the COVID-19 pandemic while restrictions on in-group sessions are in place. When in-person group sessions are allowed, we will slowly transition to in-person group sessions (by continuing to offer both options for some time).

Consent

All participants will be asked to consent using electronic consent. Electronic consent will be obtained from participants using the REDCap e-Consent framework developed by CAMH.

Participants invited to participate in the research study will be asked to provide Informed Consent during the pre-assessment appointment (via Webex or phone). During review of the Consent Form, trained senior lab members will explain the purpose of the research study, the treatment intervention, the risks and benefits to participating, the expected duration of subject’s participation, the subject’s responsibilities, the compensation for participating, confidentiality and privacy, and that all participation is completely voluntary. The individual obtaining consent must also indicate that if participants do choose to withdraw, it will in no way affect their relationship with the CAMH or prevent access to future services here.

Only after the Consent Form has been reviewed in great detail will participants be asked to sign and initial where appropriate. If participants do not feel as though they thoroughly understand every component of the research project, the person obtaining consent must expand on any sections that are unclear. Any and all questions should be answered during the consent process and any misunderstandings clarified.

Parent(s) will be asked to provide consent for all of the following:

- Read the information sheet and have been informed of the purpose of the research
- Consent to participate in Part 1 (pre-treatment) and Part 2 (post-treatment)
- Consent to participate in the daily questionnaires for a period of 10 days
- Consent to video and audio recording for the assessment
- Consent to contact for future research
- Consent to de-identified data being used for other research

Children will also be asked to provide assent during the initial assessment. This assent will be obtained by trained senior research members. The assent form will be read to children in the presence of their parents. Children will be given the opportunity to ask questions and asked to provide oral assent for participation. Many of the same components of the Consent Form will be captured on the Assent Form, but it is the responsibility of the senior lab member to explain it using child-friendly vocabulary. The senior research member obtaining consent/assent must document that assent was obtained orally, and fill out the REDCap assent form with the participants’ name, checking off the box “Assent obtained orally”.

In the case that a child does not wish to participate, or dissents, we would not ask the child or family to complete any of the child-related tasks (parent-child interaction tasks, child-reported scales and the three-minute speech sample). We will still offer parents the option to participate in other aspects of the study, as we do not want to limit parent opportunities because of child dissent.

All participants will be provided with a complete, signed copy of their informed consent and assent form to be used for future reference, the forms will be downloaded from REDCap and emailed to the parent(s)/caregiver(s). It is the responsibility of person completing the assessment to create this copy and provide it to the participant before the end of the pre- assessment appointment.

The PI’s may withdraw participants from the study without their consent if they feel that the study is no longer in the participant’s best interest. PI’s may also choose to withdraw participants from the study if participants express thoughts of self-harm or suicidal ideation. If this does happen, participants would not receive the study intervention for the full period described in the consent form. If participants do express thoughts of self-harm of suicidal ideation, OPTED SOP #001 will be adhered to.

Video and audio recordings will be retained until they are transferred to a computer and coded by research team members into data. Once transferred and coded, the video and audio recordings on the devices will be deleted and only saved on the secure CAMH server. The folder containing all video/audio recordings will accessible to members of the research team who have received appropriate training. Video and audio recordings files on the secure CAMH server will be retained for 25 years.

Participants are free to decline being audio/video taped. All participation in this research is completely voluntary, and if participants opt out of completing a portion, they are free to do so. If participants choose to not consent to being audio/video taped, then we will not ask the family to complete the FMSS, TMSS or Parent-Child Interaction Tasks (the only tasks that require audio and video recording). We will still offer parents the option of completing the rest of the study, as we do not want to limit accessibility due to parents declining one portion of the research. If parents initially consent to video and audio recording, but later change their mind, we will delete their recordings.

Once a participant agrees to participate in the study and signs the consent form, a research note will be generated on their medical record.

**e-Consent Framework**

Electronic consent will be obtained from participants using the REDCap e-Consent framework developed by CAMH and follow the following procedure:

Participants will be provided with a read-only copy of the ICF via REDCAP prior to conducting the consent discussion. The link may be used by participants as many times as they wish (it is not single-use). Upon clicking the link, participants will review the landing page, and continue on to the ICF text. The entire contents of the ICF will be displayed according to the current REB approved consent form, minus the signature/attestation page(s).

Informed consent will be documented using the REDCap e-Consent Framework. Following the consent discussion, the prospective participant/SDM will be sent a link to the e-consent via email or WebEx/Zoom (we will already have consent for email communication at this point). The participant will complete the e-consent and be provided with the option to download and/or email themselves the signed ICF. If email is chosen, the email will only be used for this purpose (it is not retained by REDCap).

Following the participant signature, the person conducting the consent discussion will complete the Person Conducting Consent Discussion Attestation Page PDF copies of the signed ICFs and Attestation pages will be retained in the REDCap File Repository. The research team will provide the participant/SDM with a copy of the fully signed ICF via mail and/or email, in accordance with the participant’s wishes.

Recruitment Strategy for Needs Assessment: CAMH Research Registry

We will utilize the CAMH Research Registry to search for potential participants for the anonymous Needs Assessment survey. Information listed on the Research Registry includes limited PHI of individuals consenting to be contacted for research (such as date of birth, gender, ethnicity, education, date of registration at CAMH, clinic(s) attended at CAMH, and referral diagnosis). The study coordinator will access the registry to contact participants who may meet inclusion criteria for the Needs Assessment (parents of children between the ages of 6-10 who themselves have mood difficulties). We will also advertise the Needs Assessment on the Research Registry for individuals to self-refer to participate.

Sample Size

As in any open-label pilot feasibility study, outcome data cannot be used to assess efficacy, nor to estimate sample size required for a future large-scale study, given that there is no control group to eliminate the effect of passage of time nor the effect of other interventions applied during the study period.

Total study enrollment: We will recruit 100 parents from the Mood and Anxiety Service (MAAS) at CAMH and Women’s College Program to complete the needs assessment survey. To determine objective emotional, cognitive, and behavioral markers we will recruit 80 parents (40 depressed; 40 non-depressed) and their children with EBP from MAAS, WCH and the Child Youth and Emerging Adult Program (CYEAP) at CAMH. To determine feasibility of ADAPT we aim to recruit 6 - 8 parents (and their co-parents) per group for each of 3 consecutive groups that will run over the course of 2 years at the CAMH and WCH (target n=36).

Visualizing Data: All data will be collected via REDCap, this includes scales that are used by team clinicians to make decisions about inclusion and exclusion criteria, initial measures and weekly measures to track changes. In order to automize the creation of reports we will use the Neuroinformatics Platform. Responses to the questionnaires will be automatically scored and pulled into the platform in order to visualize the data in the form of graphs and figures. The clinicians will use this tool to visualize and determine if inclusion and exclusion criteria is met. Changes in measures will be tracked and visualized throughout the group treatment, and clinicians will be able to use this tool to recognize trends and discuss strategies to improve outcomes.

Data Analysis

ANOVAs will be used to conduct quantitative between group comparisons on variables of interest on the dyadic parent-child interaction task and five-minute speech sample. Descriptive statistics from the needs assessment survey, program satisfaction questionnaires, and program tracking information (e.g., participant group attendance) will be used to examine recruitment and feasibility of the ADAPT intervention. Post-treatment interviews will be thematically analyzed for aspects of impact, satisfaction and acceptability.

References

1. Copeland, W., et al., *Configurations of common childhood psychosocial risk factors.* Journal of Child Psychology and Psychiatry, 2009. **50**(4): p. 451-459.

2. Fanti, K.A. and C.C. Henrich, *Trajectories of pure and co-occurring internalizing and externalizing problems from age 2 to age 12: Findings from the National Institute of Child Health and Human Development Study of Early Child Care.* Developmental Psychology, 2010. **46**(5): p. 1159-1175.

3. Musser, E.D., et al., *Attention-deficit/hyperactivity disorder developmental trajectories related to parental expressed emotion.* Journal of Abnormal Psychology, 2016. **125**(2): p. 182-195.

4. Maughan, A., et al., *Early-occurring maternal depression and maternal negativity in predicting young children's emotion regulation and socioemotional difficulties.* Journal of Abnormal Child Psychology, 2007. **35**(5): p. 685-703.

5. Luoma, I., et al., *Longitudinal study of maternal depressive symptoms and child well-being.* Journal of the American Academy of Child & Adolescent Psychiatry, 2001. **40**(12): p. 1367-1374.

6. Fanti, K.A., G. Panayiotou, and S. Fanti, *Associating parental to child psychological symptoms: Investigating a transactional model of development.* Journal of Emotional and Behavioral Disorders, 2013. **21**(3): p. 193-210.

7. McAdams, T.A., et al., *The relationship between parental depressive symptoms and offspring psychopathology: Evidence from a children-of-twins study and an adoption study.* Psychological Medicine, 2015. **45**(12): p. 2583-2594.

8. Pelham, W.E. and G.A. Fabiano, *Evidence-Based Psychosocial Treatments for Attention-Deficit/Hyperactivity Disorder.* Journal of Clinical Child & Adolescent Psychology, 2008. **37**(1): p. 184-214.

9. Lundahl, B., H.J. Risser, and M.C. Lovejoy, *A meta-analysis of parent training: Moderators and follow-up effects.* Clinical Psychology Review, 2006. **26**(1): p. 86-104.

10. Ludmer, J.A., et al., *Accounting for the impact of parent internalizing symptoms on parent training benefits: The role of positive parenting.* Behaviour Research and Therapy, 2017. **97**: p. 252-258.

11. Sawrikar, V. and M. Dadds, *What Role for Parental Attributions in Parenting Interventions for Child Conduct Problems? Advances from Research into Practice.* Clin Child Fam Psychol Rev, 2018. **21**(1): p. 41-56.

12. Eldridge, S.M., et al., *CONSORT 2010 statement: extension to randomised pilot and feasibility trials.* British Medical Jouranl, 2016.

13. Eyberg, S.M., et al., *Manual for the diyadic parent-child interaction coding system: Third edition*. 2009, University of Florida.

14. Peris, T.S. and S.P. Hinshaw, *Family dynamics and preadolescent girls with ADHD: the relationship between expressed emotion, ADHD symptomatology, and comorbid disruptive behavior.* Journal of Child Psychology and Psychiatry, 2003. **44**(8): p. 1177-90.

15. Harvey, P. and J.A. Penzo, *Parenting a child who has intense emotions: Dialectical behavior therapy skills to help your child regulate emotional outbursts & aggressive behaviors*. 2009.

16. Perepletchtikova, F., et al., *Randomized clinical trial of dialectical behavior therapy for preadolescent children with disruptive mood dysregulation disorder.* Journal of the American Academy of Child & Adolescent Psychiatry, 2017. **1**(56): p. 832-840.

17. Deater-Deckard, K., *Parenting and child behavioural adjustment in early childhood: A quantitative genetic approach to studying family processes and child development*. Child Development, 2017. 71: p. 468-484.

18. Prime, H., Rodrigues, M., Perlman, M., & Jenkins, J., *Cognitive Sensitivity Caregiver-Child Thin Slice Dyadic Interaction Coding*, Unpublished manual, 2015.

19. Sher-Censor, E.,. *Five Minute Speech Sample in developmental research: A review*. Developmental Review, 2015. 36: p. 127–155.

20. Recchia, H. E., Ross, H. S., & Vickar, M., *Power and conflict resolution in sibling, parent–child, and spousal negotiations*. Journal of Family Psychology, 2010. 24: pg. 605-614.
